# Back to the future in a petri dish: Origin and impact of resurrected microbes in natural populations

**DOI:** 10.1111/eva.12538

**Published:** 2017-10-17

**Authors:** Shira Houwenhuyse, Emilie Macke, Lien Reyserhove, Lore Bulteel, Ellen Decaestecker

**Affiliations:** ^1^ Aquatic Biology KU Leuven Kortrijk Belgium

**Keywords:** cross‐infection experiments, host–parasite interactions, permafrost, resurrected microbes, transplants, virulence evolution

## Abstract

Current natural populations face new interactions because of the re‐emergence of ancient microbes and viruses. These risks come from the re‐emergence of pathogens kept in laboratories or from pathogens that are retained in the permafrost, which become available upon thawing due to climate change. We here focus on the effects of such re‐emergence in natural host populations based on evolutionary theory of virulence and long‐term studies, which investigate host–pathogen adaptations. Pathogens tend to be locally and temporally adapted to their co‐occurring hosts, but when pathogens from a different environment or different time enter the host community, the degree to which a new host–pathogen interaction is a threat will depend on the specific genotypic associations, the time lag between the host and the pathogen, and the interactions with native or recent host and pathogen species. Some insights can be obtained from long‐term studies using a resurrection ecology approach. These long‐term studies based on time‐shift experiments are essential to obtain insight into the mechanisms underlying host–pathogen coevolution at several ecological and temporal scales. As past pathogens and their corresponding host(s) can differ in infectivity and susceptibility, strong reciprocal selective pressures can be induced by the pathogen. These strong selective pressures often result in an escalating arms race, but do not necessarily result in increased infectivity over time. Human health can also be impacted by these resurrected pathogens as the majority of emerging infectious diseases are zoonoses, which are infectious diseases originating from animal populations naturally transmitted to humans. The sanitary risk associated with pathogen emergence from different environments (spatial or temporal) depends on a combination of socioeconomic, environmental, and ecological factors that affect the virulence or the pathogenic potential of microbes and their ability to infect susceptible host populations.

## 1 INTRODUCTION

Microbes are ubiquitous on our planet (Ghai et al., 2011; Rodriguez‐Brito et al., 2010), which includes their presence in deep rock or sediment strata (Gadd, 2010; Kallmeyer, Pockalny, Adhikari, Smith, & D'Hondt, 2012), as well as being sequestered in ice sheets/glaciers/permafrost (Bidle, Lee, Marchant, Falkowski, & Karl, 2007). Recent work in the field of resurrection ecology (Orsini et al., 2013; Barras, 2017; papers in this special issue—in particular, Shoemaker and Lennon) has indicated that long‐dormant microbes can be revived, even after millions of years (Barras, 2017). The purpose of this review was to focus on the potential for revival of long‐dormant microbial pathogens. In particular, (i) we examined the risk of re‐emergence of ancient microbes from research facilities and ice sheets/permafrost, (ii) we discuss temporal adaptation of pathogens and hosts and the associated evolution of virulence of resurrected pathogens, and (iii) we conclude with a discussion of future avenues of research in the field of microbe/pathogen resurrection ecology, including both potential challenges and opportunities.

### 1.1 Microbes revived in the laboratory or conserved in research facilities

**Figure 1:**
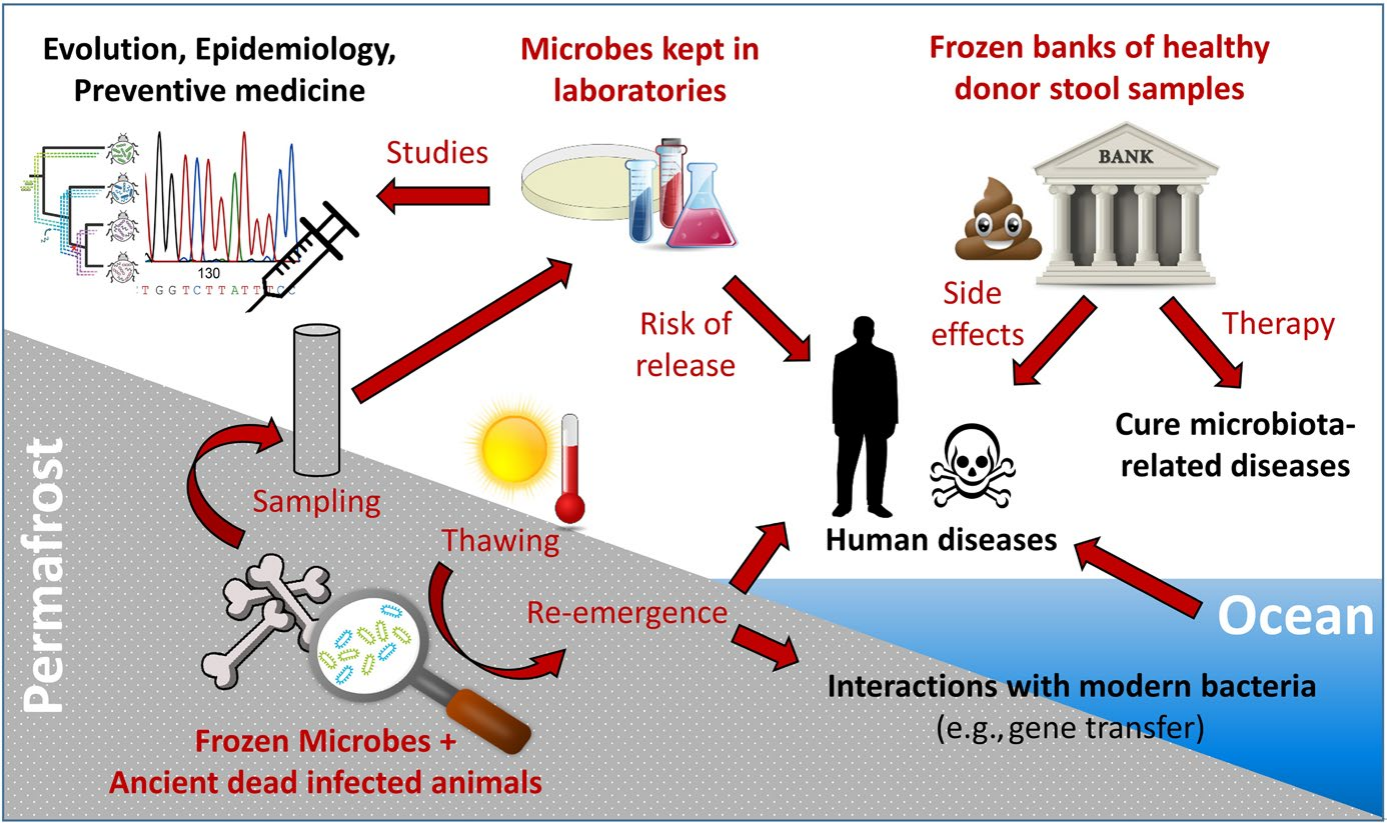
Overview of the different trajectories of how frozen pathogens or microbes can be introduced into current populations

**Figure 2:**
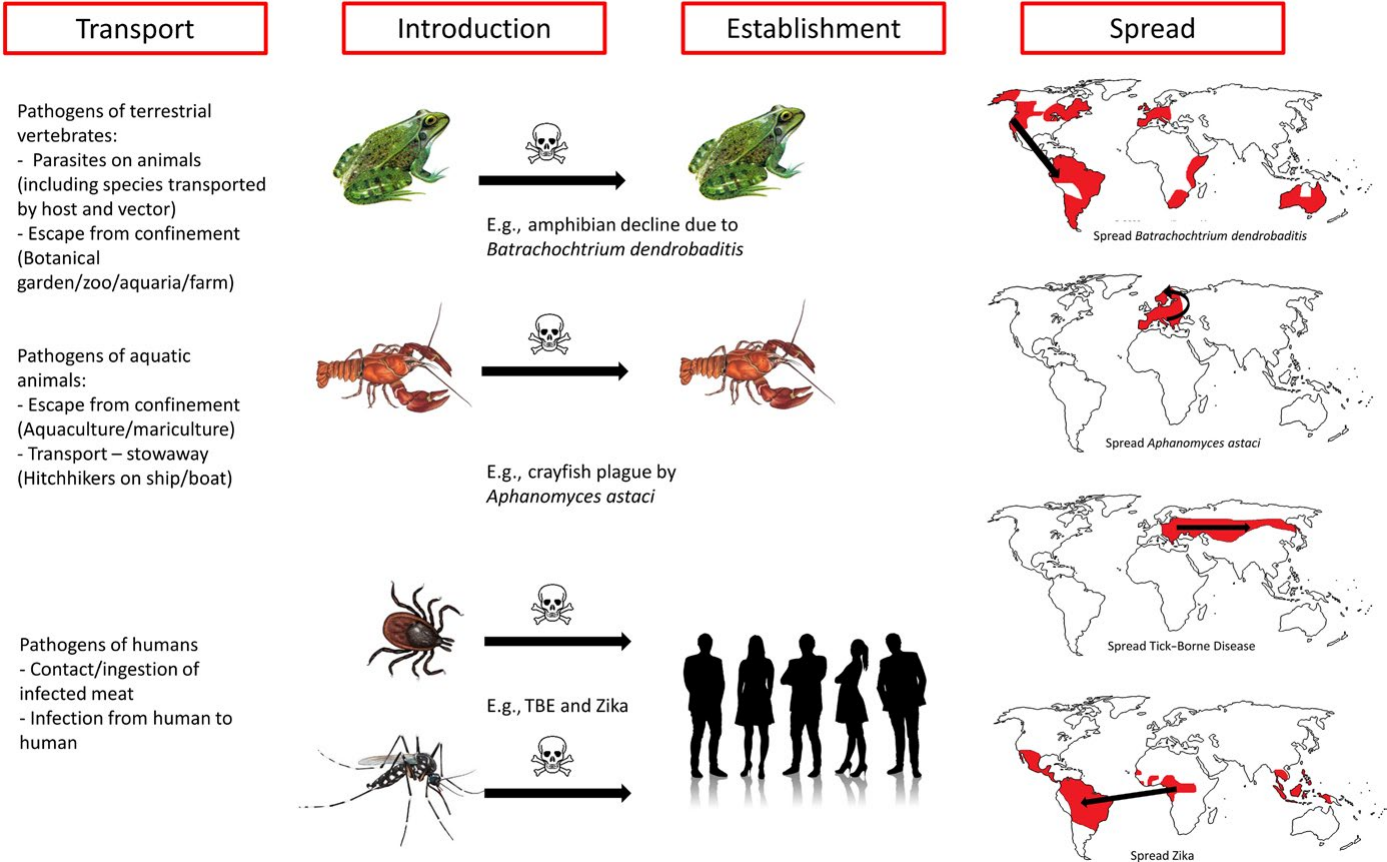
Alien pathogens can be transported and subsequently introduced through a range of pathways. Some will successfully establish and persist within hosts present within the introduced range and, furthermore, some will spread with the potential to threaten wildlife or humans

Ancient—but still potentially infectious—viruses or microbes remain widely disseminated among research and diagnostic facilities around the world. Despite the rigorous biosafety conditions that such laboratories have to respect, the risk for pathogen release into susceptible animal populations still exists (Figure 1). Although the movement of pathogens in space is probably a much higher threat than movement of pathogens over time, there are important cases to consider. In 2003, a SARS virus escaped from a level 3 laboratory in Singapore; in 2007, a biosecurity breach resulted in an outbreak of foot‐and‐mouth diseases among cattle in the United Kingdom (von Bubnoff, 2005; Hamilton, Visser, Evans, & Vallat, 2015). The risk associated with the study of ancient pathogens comes not only from frozen pathogenic strains kept in the laboratory, but also from the publication of the genome sequence of such pathogens and their potential reconstruction. For example, the genome sequence of the 1918 Spanish influenza virus, a deadly virus that killed 50 million people worldwide, was reconstructed from viral RNA retrieved from preserved tissues of victims who succumbed in 1918. A series of experiments was then conducted to study the virulence of the virus in vitro and in animal models, using viral constructs containing the 1918 genes. In stark contrast to contemporary human influenza H1N1 viruses, the 1918 pandemic virus was found to be extremely virulent, causing 100% death in mice and displaying a high‐growth phenotype in human bronchial epithelial cells (Tumpey et al., 2005). This kind of study, bringing ancient deadly viruses back to life, is still highly controversial. While some researchers point out that such studies provide key novel insights into the virus biology and pathogenesis, important information about how to prevent and control future pandemics is still lacking (Drancourt & Raoult, 2005; Taubenberger et al., 2012). The processes and associated risks of resurrected pathogens from the past can be compared with the risk of emerging and re‐emerging zoonotic diseases worldwide due to spatial invasion (Box 1) of non‐native species establishing in a new range and spreading to form new populations (Blackburn et al., 2011; Dunn & Hatcher, 2015; Hulme, 2014). Resurrected pathogens from the past and emerging pathogens due to spatial invasion are both a source of new host–pathogen interactions. In both cases, the outcome of these new interactions is unpredictable. Temporal or spatial invasion of non‐native pathogens can have devastating consequences for plants, animals, or humans (e.g., amphibian decline, crayfish plague, tick‐borne encephalitis, and Zika; see Figure 2; Box 1), but these new pathogens can also be nonvirulent to the native community and, thus, do not pose a real risk.

Box 1 **Implications of invasion biology for resurrected pathogens**

1 Despite current advances in our understanding of biological invasions, little is known about the biology of non‐native pathogens (i.e., disease‐causing pathogens including viruses, bacteria, fungi, protists, and nematodes) and their impact on biodiversity and emergence of zoonotic diseases after introduction into new regions (Hulme, 2014; Roy et al., 2016). Pathogens are frequent players in biological invasions because they are either introduced into new communities along with invading species or left behind in the ancestral range of the host, affording the host “enemy release” (such enemy release is predicted to enhance competitive ability in the new range, and this is particularly important for competitively dominant species that are limited by pathogens in their original range; Dunn & Hatcher, 2015). The introduction of non‐native pathogens can lead to new host–pathogen or pathogen–pathogen interactions (Dunn & Hatcher, 2015). The consequences of exposure of non‐native pathogens to the community are difficult to predict (Boissier et al., 2016). To date, attention has largely focused on major diseases of humans, domesticated livestock, and cultivated plants. The spread of non‐native pathogens, which affect wildlife, has received less attention despite the magnitude of their potential effects on endangered species, ecosystems, and ecosystem services (Roy et al., 2016). Predictions of future risks have focused on how climate change might shift the distribution of hosts and/or vectors, alter the timing of their life cycles, and subsequently facilitate the establishment of new host–pathogen combinations which can lead to atypical disease scenarios (Hulme, 2014). The following are a few examples of non‐native pathogens that have entered a native community and have devastating effects on biodiversity (Figure 2). The worldwide amphibian decline is driven by environmental change and ecological novelty including habitat loss, hunting, environmental trade, and the emerging infectious disease chytridiomycosis (Crawford, Lips, & Bermingham, 2010; Dunn & Hatcher, 2015). This disease is caused by *Batrachochytrium dendrobatidis*, an invasive fungus infecting over 350 amphibian species (Fisher, Garner, & Walker, 2009). The spread of this fungus has been linked to several anthropogenic factors including climate change. However, the main driver appears to be the global trade of amphibians that act as reservoirs from which the disease may spread into wild populations (Dunn & Hatcher, 2015). Another example of the devastating effects of non‐native pathogens on a native community is the crayfish plague. The crayfish plague, caused by the fungus *Aphanomyces astaci*, was introduced in Europe by the invasive signal crayfish (*Pacifastacus leniusculus*). This introduction has led to the extinctions of the local populations of the native white‐clawed crayfish (*Austropotamobius pallipes*) (Dunn & Hatcher, 2015; Holdich & Poeckl, 2007). Although pathogens can cause local extinction of new hosts in the new range, they often rely on the original reservoir (i.e., host) for their persistence. For example, an outbreak of crayfish plague in Ireland, which does not harbor the signal crayfish, led to rapid local decline of the native crayfish, but the fungus also died out (Dunn & Hatcher, 2015; Reynolds, 1988). Besides threats to animals, also humans are at risk when non‐native pathogens enter the native community. Tick‐borne encephalitis (TBE) is a human viral infectious disease caused by the tick‐borne encephalitis virus (TBEV). The virus is transmitted by the bite of infected ticks (mainly *Ixodes ricinus* in Europe), but humans can also acquire infection by consumption of contaminated unpasteurized dairy products. A number of biotic (e.g., host species and diversity) and abiotic (e.g., temperature, rainfall, humidity) factors may influence the presence of infected ticks and contribute to an increase in the incidence of TBE in Europe during the last decades (Heylen, Tijsse, Foncille, Matthysen, & Sprong, 2013). Another potential threat for humans is mosquito‐borne diseases*. Aedes aegypti* is a mosquito that acts as a vector for viruses, for example, causing Zika, dengue, and chikungunya. Over a million people die each year from these diseases. The changing distribution of vectors and vectored pathogens is a new global health threat (Medlock et al., 2012; Singer, 2017). Although the mosquito is of African origin, it has dispersed to tropical and subtropical areas outside of Africa. Changing climate has facilitated its dispersal to new areas that were previously uninhabitable. This mosquito is now more widely dispersed than at any point in the past and will spread rapidly throughout the world in the near future, as the planet continues to warm (Khormi & Kumar, 2014; Singer, 2017).

Over the past few years, a new set of microbes frozen in time has emerged in laboratories: frozen banks of healthy donors’ gut microbes that are used in therapeutic fecal microbiota transplants (FMT). Studies on the role of gut microbes in human health are currently booming, revealing strong associations between gut microbiota dysbioses and diverse diseases, from inflammatory bowel disease and obesity to Parkinson's and Alzheimer's diseases (Belkaid & Hand, 2014). New therapies, based on fecal transplants (i.e., the administration of fecal material from a donor into the gastrointestinal tract of a recipient, via nasogastric tube, enema, colonoscopy, or oral capsules, to change the recipient's microbial composition), are now envisaged to prevent or cure such diseases (Gupta, Allen‐Vercoe, & Petrof, 2016; Hamilton, Weingarden, Unno, Khoruts, & Sadowsky, 2013). While FMT were initially performed with fresh fecal slurries, they now also successfully use standardized, partially purified, and frozen fecal microbiota (Hamilton et al., 2013). FMT proved, for instance, to be a highly effective therapy for recurrent *Clostridium difficile* infections, whose incidence and severity have increased markedly since the 1990s, with frequent failure of standard antibiotic treatments (Hamilton et al., 2013; Weil & Hohmann, 2015). Despite their efficiency and their high therapeutic potential, FMT should be used with caution, as we still have little perspective on their long‐term effects (Weil & Hohmann, 2015). Beyond the evident risk of pathogens transmission, which can be limited by an attentive screening of stool samples (Gupta et al., 2016; Weil & Hohmann, 2015), FMT may have unexpected side effects, such as the stimulation of chronic diseases (e.g., obesity, diabetes, and atherosclerosis) or behavioral disorders in the recipient patient (Gupta et al., 2016). Indeed, evidence has accumulated that the gut microbiota is a complex community that interacts with numerous aspects of host physiology and behavior, including processes once thought to depend mainly on the host genetic program, such as development, immunity, metabolism, and the functioning of the brain (Macke, Tasiemski, Massol, Callens, & Decaestecker, 2017). In both mice and humans, the gut microbiota structure was shown to differ between lean and obese individuals (Sommer & Bäckhed, 2013; Turnbaugh et al., 2006), and reciprocal FMT between monozygotic twins discordant for their body mass index revealed the transmissibility and reversibility of the obese phenotype (Ridaura et al., 2013). Germ‐free mice receiving the stool from an obese donor developed greater adiposity than those colonized with a “lean” microbiota (Ridaura et al., 2013; Turnbaugh et al., 2006), revealing that FMT can have a huge impact on the metabolism of the recipient host. Transplant studies further highlighted a role for the gut microbiota in modulating stress responses and behaviors related to psychiatric disorders, such as anxiety and depression (Dinan & Cryan, 2015). Such effects thus need to be accounted for when choosing the donors for FMT. To illustrate this, Alang and Kelly (2015) recently reported a case of a woman successfully treated with FMT for recurrent *Clostridium difficile* infection, who developed new‐onset obesity after receiving stool from a healthy, but overweight donor. Other factors, such as genetics, ethnicity, and age, may also be important to consider when choosing a donor. Indeed, there is evidence that host genetics influence the structure of the gut microbiota (Goodrich et al., 2014) and that human populations from different countries, with different environments and lifestyles, have differences in the functional structure of their gut microbiome. The structure of the gut microbiota also varies with age, and it has been hypothesized that specific microbial genes that are beneficial early in life may be harmful later in life, as exemplified by the bacterium *Helicobacter pylori* which improves control of infection and allergy early in life but promotes atrophy and oncogenesis later on (Cho & Blaser, 2012; Lin & Koskella, 2014). When performing FMT, some donors may thus be more compatible than others with a given recipient patient. Taken together, these data suggest that the choice of the donor is crucial, which questions the appropriateness of universal frozen stool banks.

### 1.2 Ancient microbes emerging from the permafrost

Next to the threat that is present in laboratories, a second risk is present in natural ecosystems, given that our planet is in a time of climate change. These changes can be observed in the atmosphere (increasing temperatures), the ocean (ocean warming, increasing salinization, acidification, and rising sea levels), and the cryosphere (loss of ice mass and snow cover). In general, climate change poses potential risks to people, society, economy, and ecosystems. The greatest increase in temperature has been observed in areas of mid‐ and high latitudes, as arctic temperatures are rising rapidly, being twice the global rate (Schuur & Abbott, 2011; Slenning, 2010). Natural microbial populations in the permafrost are thus shaped by current and future environmental challenges and interactions. In particular, a historical factor that may induce future challenges is present via the resurrection of dormant propagules and viable vectors (Figure 1). As the permafrost serves as a natural bank, it contains a variety of dormant propagules (seeds, eggs, cysts, or spores from plants and invertebrates) and other viable vectors (bacteria, viruses). This may increase the risk of infections (Revich & Podolnaya, 2011; Revich, Tokarevich, & Parkinson, 2012). Over the past few years, there has been increasing evidence that the permafrost is a gigantic reservoir of ancient microbes or viruses that may come back to life if environmental conditions change and set them free again. The amount of microorganisms trapped in permafrost which remains viable can range up to 10^8^ cells/g of dry soil (Vorobyova et al., 1997). The thawing of the permafrost increases the resurrection of different dormant vectors, such as bacteria which can remain viable for several million years (Vishnivetskaya, Kathariou, McGrath, Gilichinsky, & Tiedje, 2000). For example in 2014, a viable specimen of a giant virus, named *Pithovirus sibericum*, was found in a 30,000‐year‐old ice core harvested from Siberian permafrost, and revived in the laboratory. Interestingly, this virus was found to be still infective to its natural amoeboid host (Legendre et al., 2014). Similarly, Ng et al. (2014) recovered and characterized two viruses preserved in 700‐year‐old caribou feces frozen in a permanent ice patch. One of these viruses is a distant relative of the geminiviruses, a well‐known group of plant viruses, whereas the second one is related to a group of insect RNA viruses pathogenic to beneficial arthropods, such as honeybees, as well as to insect pests of medical and agricultural importance. Remarkably, these viruses were still intact and remained infectious after being 700 years in ice. There is thus a risk, especially given that the Russian Arctic is particularly affected by climate change. Especially, the plant virus was shown to be infective to the modern plant *Nicotiana benthamiana*, a relative of tobacco that is native to northern Australia and is thus clearly not the natural host of this virus (Holmes, 2014; Ng et al., 2014). Such results indicate that potentially infectious pathogens might be released from ancient permafrost layers exposed to thawing with potential consequences for human, animal, and plant populations. In addition, the rich mineral resources and oil reserves of the arctic regions are under increasing pressure for their industrial exploitation (i.e., mining and drilling; Revich et al., 2012; Legendre et al., 2014). Such events will result in flooding and disruption of soil, which may release bacterial spores or viruses onto the surface soil and vegetation which would then be consumed by grazing animals, also increasing the risk of infection in humans who come into contact with infected animal products (under cooked meat, hides, bone; Revich et al., 2012). Although the risk posed by potential pathogens trapped in ice is low compared to the normal horizontal spread of contemporary viruses among host populations, and the warmer temperature associated with ice melting may partly degrade viral nucleic acids (Holmes, 2014), these pathogens are not exempt from posing future threats to human and animal health (Legendre et al., 2014). In Russia, epizootic cycles of anthrax which caused the death of 1.5 million deer between 1897 and 1925 have resulted in more than 13,000 burial grounds containing the carcasses of infected animals. With ice thawing, these carcasses may reappear, resulting in frequent anthrax outbreaks among cattle and reindeer, also infecting humans who process infected animal products or ingest improperly cooked infected meat (Revich et al., 2012). In 2016, the indigenous peoples in the Yamal‐Nenets Region (Russia) were victims of anthrax and needed to be hospitalized, while the herd of reindeer they use as food source had perished from either the infection or extermination by the Russian Defense Ministry. Thousands of people were forced to relocate, while others were quarantined. This outbreak is thought to stem from the thawing burial grounds of reindeer carcasses dead from an anthrax outbreak 75 years ago.

The community DNA immobilized in permafrost represents an important reservoir of genes that may potentially be acquired by extant microbes upon thawing through lateral gene transfer. Bidle et al. (2007) performed a metagenomic analysis of community DNA found in 100‐ka‐ to 8‐MA‐old ice samples and revealed many diverse orthologs to extant metabolic genes, and some microbes isolated from the same samples were even able to grow in the laboratory. Their analysis suggests that melting of polar ice in the geological past may have provided a conduit for large‐scale lateral gene transfer, potentially scrambling microbial phylogenies and accelerating the tempo of microbial evolution (Bidle et al., 2007). Remarkably, genes encoding resistance against natural or modern semisynthetic antibiotics, as well as mobile elements participating in their horizontal transfer, were found in bacterial strains isolated from Siberian and Antarctic permafrost grounds, dating from 5,000 to 30,000 years ago (Perron et al., 2015; Petrova, Gorlenko, & Mindlin, 2009; Petrova, Kurakov, Shcherbatova, & Mindlin, 2014). Many of these resistance genes were highly similar to resistance genes found in pathogenic bacteria today, confirming the hypothesis that the antibiotic resistance genes of clinical bacteria originated from environmental bacteria. Taken together, these results support the hypothesis that a reservoir of resistance genes existed in a range of bacteria species prior to the anthropogenic use of antibiotics and contribute to a growing body of evidence demonstrating that antibiotic resistance evolved alongside antibiotic production in the natural environment (Perron et al., 2015; Petrova et al., 2014). These results also support the growing body of evidence that nonpathogenic environmental organisms, including those present in the permafrost, are a reservoir of resistance genes that have the potential to be transferred into pathogens, and thus greatly affect the evolution of multidrug‐resistant bacteria in clinical settings (Bhullar et al., 2012).

To make it more complex, there are also microbes that protect their hosts from pathogenic infections. The interaction between these “defensive” microbes and pathogens coevolves within host populations. Ford, Wiliams, Paterson, and King (2017) experimentally coevolved a microbe with host‐protective properties (*Enterococcus faecalis*) and a pathogen (*Staphylococcus aureus*) within nonevolving *Caenorhabditis elegans* host populations. They found that after 10 passages, all replicate pathogen populations were locally adapted in time to the defensive microbe, while the defensive microbe was locally maladapted. This pattern is parallel with host–pathogen coevolution studies which have found that pathogen local adaptation is more commonly identified than host local adaptation because pathogens have faster evolutionary responses to reciprocal adaptation than hosts (due to shorter generation time, larger populations sizes, and higher rates of migration; Ford et al., 2017; Kawecki & Ebert, 2004). By performing time‐shift experiments, Ford et al. (2017) showed that both the defensive microbe and the pathogen peaked in fitness against antagonist populations recently experienced in evolutionary history. Defensive microbes from the future were significantly better at suppressing pathogens than defensive microbes from the past, while future pathogens were significantly better at suppressing defensive microbes than pathogens from the past. The fitness of a focal species against their recent enemy is expected to be higher than against their current enemy as the current enemy has begun to respond to the adaptive changes of the focal species. This shows that interactions between non‐native pathogens (emerging from a different location or from a different time) and native hosts can be very complex. Besides pathogens, defensive microbes can also enter the community and alter the interactions between pathogen and host. This can be in a positive way by reducing the virulence of the pathogen, but it can also be in a negative way by increasing the virulence of the pathogen, depending on the time lag between the pathogen and the host (Dybdahl & Lively, 1998; Ford et al., 2017; and see further details in the temporal adaptation section below).

### 1.3 Factors determining the risk of emerging infectious diseases from ancient pathogens

The health risk associated with pathogens emergence depends on a combination of socioeconomic, environmental, and ecological factors that affect the virulence or the pathogenic potential of microbes and their ability to infect susceptible host populations. The emergence and transmission of an infectious disease typically follows a pathway that involves a reservoir (i.e., the environment, a vector, or a secondary host) to contact a native host (i.e., human or animal species). The risk of disease emergence thus greatly depends on the rate of contact and/or spillover between reservoirs and the native host (Lambin, Tran, Vanwambeke, Linard, & Soti, 2010; Nii‐Trebi, 2017). In zoonoses, which represent the majority of emerging infectious diseases, contact between wildlife reservoir species and livestock or humans creates interfaces that might be important for the transmission of pathogens. Many synanthropic species (i.e., species, animals, or plants that live near humans), such as rodents, birds, bats, and certain other mammal species, have been shown to carry zoonotic pathogens and in some cases act as reservoir hosts for these pathogens. Anthropogenic pressures associated with urbanization often bring these species into closer contact with livestock and humans and thus favor disease emergence (Hassell, Begon, Ward, & Fevre, 2017). Another important factor influencing human–livestock–wildlife contact is livestock husbandry. Informal livestock raising is commonplace in African cities and is often characterized by low biosecurity and mixed‐species livestock being kept in close proximity to humans. Evidence from recent zoonotic emergence events in Asia (such as avian influenza viruses; World Health Organization Global Influenza Program Surveillance Network, 2005) and the circulation of relatively stable zoonoses (such as bovine tuberculosis; Gortazar et al., 2011) implicate a role for livestock acting as bridge hosts, epidemiologically linking wildlife and humans. Sociodemographic factors, such as population density, migration, trade, conflicts, social instability, or access to clean water, also have important impacts on the transmission of pathogens and vector dynamics and largely influence the epidemiology of infectious diseases (Hassell et al., 2017; Nii‐Trebi, 2017). The chain of infection further depends on the vulnerability of host populations. Not all conspecifics are competent hosts for a given pathogen (Bento et al., 2017), and “dead‐end” hosts can play a role in regulating infection. Important factors influencing host susceptibility to infection are age and gender, but also immunocompetence and immunological history (Hassell et al., 2017). If ancient pathogens, such as the 1918 influenza virus, were to re‐emerge, it may not have the same consequences as the past pandemics. Indeed, in the case of the 1918 influenza virus, for example, most people now have some immunity to the 1918 virus because subsequent human flu viruses are in part derived from it. And, in mice, regular flu vaccines and drugs are at least partly effective against an infection with reconstructed viruses that contain some of the genes from the 1918 flu (von Bubnoff, 2005).

## 2 RESEARCH AVENUES OF RESURRECTION ECOLOGY

### 2.1 Temporal adaptation of pathogen infectivity and host susceptibility

To fully comprehend the outcome of new host–pathogen associations, it is important to obtain insight into the mechanisms underlying host–pathogen interactions at several spatial and temporal scales of adaptation (Penczykowski, Lane, & Koskella, 2016). Examination of patterns of host–pathogen adaptation across space has typically been considered a more rapid approach for inferring coevolutionary dynamics over time (Burdon & Thrall, 2014; Gandon, Buckling, Decaestecker, & Day, 2008; Penczykowski et al., 2016). This is, however, not always straightforward, given the time lag differences that are hidden in such associations (Gandon et al., 2008).

Differences in the susceptibility of the host and infectivity of the pathogens induce strong reciprocal selective pressures and often result in an escalating arms race of adaptations over time (Decaestecker, De Gersem, Michalakis, & Raeymaekers, 2013; Decaestecker et al., 2007; Futuyama & Agrawal, 2009; Schmid‐Hempel, 2011). One of the most important aspects of adaptation in two antagonistic species is the relative rate at which they (co)evolve. As pathogens are typically smaller than the hosts, have a much shorter generation time, and have larger populations sizes and higher rates of migration, they often have a higher evolutionary potential (Greischar & Koskella, 2007; Kawecki & Ebert, 2004). This has led to the conventional wisdom that pathogens should be locally adapted more often than their hosts. However, pathogens are often asexual while their hosts reproduce sexually, which can accelerate host response to pathogens. This genetic variation in the host is the fuel for reciprocal coevolutionary dynamics in host resistance and pathogen infectivity (Decaestecker et al., 2007, 2013; Kawecki & Ebert, 2004). It is important to note that there are two types of host–pathogen coevolutionary dynamics: (i) arms race dynamics, which are a series of selective sweeps of new mutations, and thus a slow process, and (ii) negative, frequency‐dependent coevolutionary dynamics, which is a much faster process, involving the cycling of alleles that have an advantage when rare (Dybdahl & Lively, 1995; Gandon et al., 2008). This negative, frequency‐dependent selection by pathogens results in cyclic dynamics of host and pathogen genotypes, each with their own resistance and infectivity, the so‐called Red Queen dynamics (RQDs). These coevolutionary RQDs are associated with a status quo in infectivity over time and have been suggested to play a role in widespread biological phenomena such as the evolution and maintenance of sexual reproduction, and to shape the structure of natural populations and communities (Decaestecker et al., 2007; Maynard‐Smith, 1989; Wood et al., 2007).

**Figure 3:**
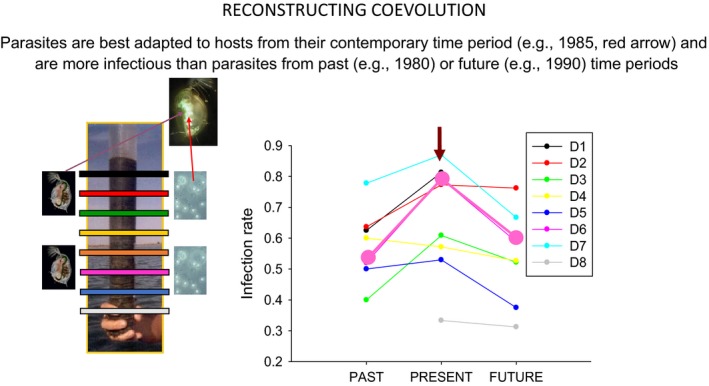
Illustration of a “resurrection ecology” approach to reconstruct Red Queen coevolutionary dynamics of Daphnia–pathogen interactions (Decaestecker et al., 2007)

Due to the timescale required to witness long‐term changes in genotype frequency and adaptation, it is challenging to study long‐term effects of environmental change on evolutionary and ecological patterns. One way to circumvent this problem is by using a “resurrection ecology” approach (e.g., the water flea—*Daphnia*—and its parasites), where resting stages are hatched out of different sediment layers from a sediment core, representing different time points in a specific pond (Angeler, 2007; Decaestecker, Declerck, De Meester, & Ebert, 2005; Orsini et al., 2013). These “resurrected” populations can then be used in time‐shift experiments (Brockhurst & Koskella, 2013; Gaba & Ebert, 2009) where pathogens from one particular time point are exposed to a host from a past, contemporary, or future time point. By analyzing these particular interactions, the researcher can directly observe the long‐term dynamics of host–pathogen coevolution. Basically, if the fitness of a contemporary population (e.g., the host) is higher than the fitness of the past population, but lower than that of the future population when exposed to a fixed population of the antagonist (e.g., the pathogen), this indicates that the host population has responded to selection pressures induced by the pathogen over a given time point (Buckling & Rainey, 2002; Decaestecker et al., 2007; Koskella, 2013). In these experiments, one essential point to consider is the time frame to test for coevolution, especially in the context of rapid evolutionary change (Decaestecker et al., 2007, 2013; Figure 3). It is therefore best to perform time‐shift experiments across time points spanning the predicted window over which coevolution is expected to occur (Penczykowski et al., 2016). This information can then be used to expose the resurrected populations to a reconstruction of their past, contemporary, and future environments, which provides useful information about patterns of adaptation of these populations toward their respective environments. If we expect cycling of host and pathogen alleles as hypothesized by Dybdahl and Lively (1995), we can imagine the infectivity of combinations involving pathogens from the past and hosts from the future. Given the time lag of pathogen adaptation to the host, combinations of near‐future pathogens with past hosts are assumed to be the most infective (Gandon et al., 2008). It is thus relatively unlikely that resurrected pathogens from the past are the best adapted strains to recent (future) hosts, given that they will need some time to adapt to the relatively new host. Nevertheless, occasionally, this may develop rapidly and/or perfect host–pathogen matches can occur resulting in high pathogen infectivity; however, this is difficult to predict.

Recently, a powerful method wedding synthetic biology and ancestral sequence resurrection with experimental evolution has been developed to study evolution at the molecular level in bacterial genomes (Kacar, Ge, Sanyal, & Gaucher, 2017). This paleo‐experimental evolution approach consists of resurrecting an ancient gene, removing the modern form of the gene from an extant organism (e.g., *E. coli*), and then inserting the ancestral form into the extant organism. Laboratory evolution is then used to examine the adaptive potential of the hybrid genome. As an example, a 700‐million‐year‐old inferred ancestral variant of tufB, an essential gene encoding elongation factor Tu, was engineered and inserted into a modern *E. coli* genome in place of the native tufB gene. This replacement was detrimental to *E. coli* fitness, mainly due to reduced protein dosage. Interestingly, experimental evolution allowed bacteria to restore fitness, through accumulation of mutations in the promoter of tufB, leading to increased expression of the ancient EF‐Tu protein. These mutations may constitute the most facile pathway for compensatory genetic changes, particularly for highly conserved proteins. Such paleo‐experimental evolutionary studies allow a rewind and replay of the evolutionary history of ancient biomolecules in the laboratory and may provide a deeper understanding of the roles that contingency and determinism play in evolutionary processes (Kacar et al., 2017).

### 2.2 Virulence evolution in resurrected host–pathogen interactions

Due to climate change and other types of anthropogenic change, ancient pathogens can re‐emerge. This can lead to spatial or temporal mismatches between antagonists (host and pathogens), resulting in host shifts, and the rapid spread of disease across susceptible host populations not historically exposed to particular pathogens (Penczykowski et al., 2016). In addition, it can lead to altered virulence effects. To estimate the effect of emerged pathogens (from different locations or different time points) in natural populations, it is thus important to estimate their virulence upon introduction. There are three phases in the adaptation of a pathogen to a new host: (i) accidental infections, (ii evolution of virulence soon after successful invasion, and (iii) the evolution of optimal virulence in a coevolutionary interaction between the host and the pathogen. These three phases show how the virulence of the pathogen evolves. In the first phase (accidental infections), pathogens infect and cause disease in a host that is not part of the normal transmission route but is a dead end for the pathogen. These accidental infections could occur when pathogens are resurrected from the permafrost or when pathogens expand their infection area (e.g., when pathogens are introduced in a new area via exotic species, or via air travel or shipping). Pathogens tend to be locally and temporally adapted to their co‐occurring hosts, but when pathogens from a different environment or different time enter the host community, the level of virulence in these new host–pathogen interactions is unpredictable (Levin & Svanborg‐Edén, 1990). Virulence in novel hosts does not represent an equilibrium for pathogen fitness, and it is unlikely to be associated with high transmissibility (Ebert & Bull, 2008). In most cases, infectivity and virulence are, on average, higher in established associations than in novel ones (Ebert, 1994; Lively, 1989). There can, however, be considerable variation around this average (Ebert, Zschokke‐Rohringer, & Carius, 1998; Kaltz, Gandon, Michalakis, & Shykoff, 1999): A novel combination may be avirulent on average, but may occasionally be highly virulent (Ebert, 1994). To enter the second phase, an accidental infection of one individual spreads to another host, then another, forming a continual transmission chain that successfully invades the new host population. Initially, this spread will constitute an epidemic, in which the number of infected hosts increases. Phase 2 also applies to the origin of novel variants of a pathogen in the old host. These are extreme mutants of existing pathogens that are able to start epidemics (Ebert & Bull, 2008). The invading pathogen will generally not be well adapted to the new host, and consequently, there will be rapid evolution of the pathogen and probably the host. Virulence may be far from optimal in this initial establishment in the new host, but their advantage outweighs other suboptimal traits, so that they can spread nonetheless. After persisting for some time in a new host population, the pathogen should approach equilibrium virulence. This is the third phase in the adaptation of a pathogen to a host, the evolution of optimal virulence. The pathogen may then reach a selection boundary, in which trade‐offs among the pathogen's various fitness components constrain further evolution of virulence and transmission (Anderson & May, 1982; Ewald, 1980). Anderson and May (1982) found evidence for a trade‐off between pathogen‐induced host mortality (i.e., virulence) and host‐induced pathogen mortality (i.e., host recovery). The trade‐off model is versatile and makes it possible to predict changes in optimal virulence for different conditions. The trade‐off virulence model predicts that transmission‐stage production and host exploitation are balanced, such that the pathogen's lifetime transmission success is maximized. Killing the host after an intermediate time period results in maximal lifetime transmission success (Jensen, Little, Skorping, & Ebert, 2006). This trade‐off model makes it possible to predict what level of virulence will evolve when new host–pathogen interactions are established (e.g., when pathogens arise from a different environment or time).

During the coevolutionary arms race, hosts evolve to reduce the damage that pathogens cause to their fitness (Ebert & Bull, 2008). Some components may impact both partners, while others may not. Therefore, it is important to specify the bases of virulence when discussing host–pathogen coevolution (Ebert & Bull, 2008). Masri et al. (2015) used an experimental design consisting of five distinct evolution treatments: (i) host control, where the host adapted to general laboratory conditions in the absence of a pathogen, (ii) host one‐sided adaptation, where the host adapted to an ancestral pathogen, (iii) host–pathogen coevolution, where both the host and pathogen coadapted to their continuously coevolving antagonist, (iv) pathogen one‐sided adaptation, where the pathogen adapted to an ancestral host population, and (v) pathogen control, where the pathogen adapted to general laboratory conditions in the absence of a host. This study showed that high virulence was specifically favored during host–pathogen coevolution rather than pathogen one‐sided adaptation to a nonchanging host or to an environment without the host, warning us to increase monitoring and surveillance and take concerted actions to deal with new host–pathogen interactions.

One of the oldest and most successful uses of virulence evolution has been technological: to create attenuated live viruses. Live vaccines are strains of a formerly pathogenic organism that have evolved to become avirulent (Ebert & Bull, 2008). Before the advent of genetic engineering, the standard method for developing a live vaccine was to adapt a virulent pathogen to grow in culture. As the pathogen evolved to grow better in culture, it also often evolved to grow poorly in the normal host, where its virulence was therefore reduced (Ebert, 1998). This outcome is a trade‐off between the ability to grow under one set of conditions and that of another (local adaptation). This method did not always succeed, but it was robust enough to succeed in many cases. The low virulence of attenuated vaccines can be reversed by evolution. If the vaccine strain is again allowed to transmit between hosts, natural selection may promote the evolution of variants that grow better and reacquire high virulence (Ebert & Bull, 2008).

The study of host–pathogen interactions across spatial and temporal scales is increasingly important as there is increasing evidence that these scales are changing due to human‐mediated factors including climate change, habitat fragmentation, and increased dispersal (Alexander, Mauck, Whitfield, Garrett, & Malmstrom, 2014; Alitzer, Ostfeld, Johnson, Kutz, & Harvell, 2013; Opdam & Wascher, 2004; Penczykowski et al., 2016). These and other types of anthropogenic change may lead to spatial or temporal mismatches between antagonists, resulting in host shifts and the rapid spread of disease across susceptible host populations not historically exposed to particular pathogens (Penczykowski et al., 2016). For instance, an increased level of eutrophication (i.e., nutrient “pollution”) in time can lead to increased pathogen prevalence and virulence, which may promote evolution toward elevated virulence (Aalto, Decaestecker, & Pulkkinen, 2015; Decaestecker et al., 2007; Forde, Thompson, & Bohannan, 2004; Johnson et al., 2010). Lake sediments serve as archives of environmental change, where paleolimnological methods can be used to reconstruct changes in an elemental context (Bennion, Fluin, & Simpson, 2004; Battarbee, Anderson, Jeppesen, & Leavitt, 2005; see Burge et al., this issue). It is estimated that if environmental nutrient enrichment or increased temperature is translated into higher population sizes, then increased host–pathogen interactions, in terms of within‐host competition and pathogen transmission, may intensify the coevolutionary arms race and virulence outcome between hosts and pathogens (Aalto et al., 2015; Reyserhove et al., 2017). Together, the analyses of stratified egg banks are a key instrument to link evolutionary dynamics in natural populations with environmental change (Frisch et al., 2013, 2016; Hairston et al., 1999, 2001). Variation in both biotic and abiotic factors strongly shapes host–pathogen interactions and the mode of coevolution, so when pathogens from a different location or different time period encounter a new host, environmental conditions in the old and new environment will affect the risk.

## 3 CONCLUSIONS AND FUTURE AVENUES

With arctic temperatures rising twice as fast compared with the global average rate (Schuur & Abbott, 2011; Slenning, 2010) and the increasing thawing of permafrost and ice sheets (Revich et al., 2012), many questions remain concerning the trapped microbes which can possibly re‐emerge and the microbes that expand their current habitat due to the changing climate. Additionally, ancient pathogenic material is maintained in laboratory environments, and if not secured correctly, an (un)intentional outbreak can occur. The question remains: How great is the chance of re‐emerging epidemics/pandemics and what will be the overall effect? Thus, it is essential to study these new interactions with (re‐)emerged pathogens from the past (or from a different environment) as they are unpredictable. Some insights can be obtained from long‐term experiments using a resurrection ecology approach (Decaestecker et al., 2007; Gaba & Ebert, 2009). These long‐term studies based on time‐shift experiments are essential to obtain insight into the mechanisms underlying host–pathogen coevolution at several spatial and temporal scales and possibly make predictions. Complicating the host–pathogen coevolution are defensive microbes that protect the host against the virulence of the pathogen. These defensive microbes can emerge from a different location or from a different time and alter the host–pathogen interaction in a positive or negative way depending on the time lag between the pathogen and the host (Dybdahl & Lively, 1998; Ford et al., 2017).

Melting of permafrost and ice sheets will enable the trapped microbes to flood into the oceans, which could speed the process of evolution. As bacteria can easily trade genes by recombination, ancient resurrected bacteria can mix with current ones which can introduce new traits into the current population (Klassen & Foght, 2011). Next to genotype mixing and gene transfer, the flooding of these masses of microorganisms in the ocean can also have potential climate impacts. As the melting of permafrost and ice sheets is accompanied by the reintroduction of a huge load of carbon and organic matter, this could trigger a growth burst of bacteria (Smith & Bonnaventure, 2017). One possible hypothesis is that these boosted bacteria would use up all the oxygen in the water which could destroy fish habitats and exacerbate ocean dead zones that are already occurring from other causes (Diaz & Rosenberg, 2008). A possible additional avenue is utilizing stored or published ancient microbes for means of bioterrorism, which is the deliberate release of pathogens to induce harm or death in plants, animals, and even humans (Santana et al., 2016).

To limit hazardous events of unintentional bacteria and viral laboratory outbreaks, international efforts should focus on ensuring that all remaining stocks of infectious material are destroyed or stored safely in a minimum number of approved high‐containment facilities (Hamilton et al., 2015). Next to this, and upon a high and emerging risk of infectious diseases due to resurrected or frozen pathogens, management strategies can be undertaken. If there is a threat of spillover from wildlife to humans and subsequent spread of infection, four major strategies can be followed: (i) reducing disease prevalence in reservoir hosts, for instance, through vaccination baits, which have been successfully used to eliminate rabies from several European countries, (ii) reducing contact rate between humans and wild animals, for example, by limiting the proximity between humans and wildlife, (iii) lowering the probability of infection, when contact is unavoidable or unpredictable, through vaccination, as illustrated by human dengue vaccine which provides cross‐protection against sylvatic dengue viruses circulating in nonhuman primates, and (iv) adopting regional control strategies (including isolation of infected populations, dispatching of medical personnel and aid, and enhanced border control) to prevent disease transmission across borders when spillover does occur (Johnson, de Roode, & Fenton, 2015).

However, the resurrection of ancient microbes does not necessarily have to have a negative outcome. Studies on resurrected microbes can provide key insights into historical reconstruction, epidemiology, and evolutionary patterns of pathogens and hosts. Molecular typing of resurrected ancient microbes provides the opportunity to reconstruct past epidemics which could contribute to a better understanding of emerging infectious diseases (Duggan et al., 2016). These new insights could add to the development of preventive measures of epidemics. Next to reconstructing past epidemics, it could also contribute to knowledge on Earth's past climate. As living bacteria can be found in ice cores from 420,000 years old (Christner, Mosley‐Thompson, Thompson, & Reeve, 2001), one could extrapolate to conditions in outer space as these microbes seem to survive for millennia in such extreme conditions (extremophiles). Additionally, ancient viruses and bacteria can provide an important bank of genes which can be implemented in vaccines, new antibacterial drugs, or even cancer‐fighting chemicals (Boston, 2017).

## DATA ARCHIVING

No data are associated with this manuscript.
